# Investigating Fireside Corrosion Behavior and Mechanism of Low-Alloy Water Wall Tube of Ultra-Supercritical Power Plant

**DOI:** 10.3390/ma18071666

**Published:** 2025-04-04

**Authors:** Yifan Ni, Weijie Weng, Zuogui Zhang, Jianning Li, Chenghao Fan

**Affiliations:** Shanghai Power Equipment Research Institute, No. 1115, Jianchuan Road, Minhang, Shanghai 200240, China

**Keywords:** power generation, water wall tube, low-alloy steel, corrosion mechanism

## Abstract

The corrosion thinning behavior and mechanism of low-alloy water wall tubes of an ultra-supercritical power plant was investigated via SEM, EPMA, XRD, TEM, and laboratory simulation experiments. Fireside corrosion was first initiated by chemical potential- and concentration-governed transportation and diffusion, sequentially facilitated by sensitization, which was observed by TEM in terms of the carbide matrix precipitation on the grain boundary, and finally accelerated by the kinetic controlled growth, leading to the final thinning behavior. Laboratory experiments revealed that the reduced atmosphere corrosion kinetic simulation followed the linear law, as well as a different corrosion scale structure layer, compared to the furnace corrosion sample; the reduced atmosphere condition in the laboratory experiment inhibited the oxidation process and layer growth. The frequent shift between the oxidizing and reducing properties of the atmosphere around the water wall tubes during boiler operation may contribute to the delaminated oxidation layer.

## 1. Introduction

Low-alloy ferritic steel, which is extensively used in the petroleum and power industry, has an affordable price and a decent strength and performance under a temperature of 450 °C [1], which makes it a suitable candidate material for water wall tubes in USC power plants. The water wall tubes are the main heating part of the boiler, which are composed of rows of pipes that surround the boiler furnace, playing a crucial role in absorbing thermal radiation and facilitating the phase transformation from water to steam. While the interior of these tubes carries flowing water or steam, the exterior directly absorbs heat from the boiler furnace. Water wall tubes inevitably encounter typical failures like burst [2], corrosion [3], and abrasive thinning [4] due to the harsh circumstances in the furnace. In addition, with the synergistic effect of the increased content of actively reducing gases plus burning low-quality coal, as well as long-term high-temperature exposure, the safety and availability of the power plant water wall tubes are unexpectedly reduced [5].

As corrosion underlies one of the major causes for failures in engineering and research, it has attracted compelling interests and produced numerous repositories. Stress corrosion cracks (SCCs) are considered to be the major failure mechanism regarding all grades of alloys in the presence of stress [6], sensitization [7], and corrosive environments [8]. Li et al. [9] reported that chloride-induced SCCs were the main root cause for the SS 304 tube failure in a gas analyzer exposed to a chloride environment at the welding influence zone. Martin et al. [10] presented the reinforced carbon steel bar failure analysis induced by SCCs in terms of the carbonation process and chloride-contaminated environment, and concluded that the corrosion crack growth was more dependent on the pH than on chloride contamination. However, an in situ synchrotron X-ray tomography study conducted by Schoell et al. [11] revealed the formation of CrCl_2_, NiCl_2_, and FeCl_2_ in the vicinity of the crack branch, and it remained unclear what role chloride-bearing compounds played in the chlorine-induced stress corrosion cracking process. In addition to the impact of the corrosive environment, sensitization plays a crucial role in facilitating the growth and development of the corrosion process. Sensitization occurs when the steel is exposed to a high-temperature circumstance for an extended period of time, along with the formation of chromium carbide precipitates around the grain boundaries, which causes the depletion chromium and makes the grain boundaries more susceptible to intergranular stress corrosion cracks (IGSCCs) [12]. This conventional sensitization theory is widely accepted and has been validated by several studies [13,14,15]. Liu et al. [16] investigated the corrosion mechanism of a 304 stainless steel geothermal tube and concluded it was initiated by SCCs, and that sensitization accelerated the corrosion process to mechanical fracture. However, recently, a three-dimensional atom probe analysis conducted by Hu et al. [17,18] revealed that intergranular cracks and the corrosion of low-chromium ferritic stainless steel was largely impacted by the Cr atom segregation and co-segregation of other solute atoms in the absence of Cr carbide precipitation in 450 °C experimental environments, which sheds a new light on the corrosion mechanism compared to conventional theory. Montero et al. [19] implemented a laboratory experiment to breakaway the fireside corrosion mechanism of the austenitic steel Sanicro 25, and concluded that the formation of chromium carbides from the γmatrix of the substrate steel played a pivotal role in the corrosion process and served as the intermediate step for the corrosion mechanism. Although the corrosion behaviors and mechanisms were extensively investigated, the fireside tube corrosion analysis for the power plant component was quite limited, and the corrosion mechanism varied remarkably in the terms of the environmental conditions, material intrinsic properties, and reaction kinetics.

This investigation was carried out during the maintenance and overhaul phase of a 660 MW ultra-supercritical coal-fired plant. The facility, under the ownership of Wuhu Power Generation Co., Ltd., is situated in Wuhu, in the southeastern region of Anhui province, China. The USC boiler was designed by BABCOCK & WILCOX BEIJING Co., Ltd., with the rated boiler outlet steam parameters set at 26.15 MPa and 603 °C. The boiler heating component is inspected annually in scheduled grade (A, B, or C, which only differs in the length of the time period) overhaul activities, and the replacement of heating bundles and components are implemented only in the grade A and B overhaul activities. Significant high-temperature corrosion of the water wall tubes and thickness thinning were found during the shutdown period. Water wall tubes totaling 288 square meters on both lateral sides of the furnace were replaced during the overhaul activities. This study analyzed the corrosion products and characterized the microstructure of the in-furnace water wall tubes. Concurrently, a laboratory-scale high-temperature fireside corrosion experiment was conducted on the SA-213 T12 steel in a reducing atmosphere. The laboratory experiment was expected to provide a comparative observation of the corrosion behavior of the furnace sample and to investigate the corrosion mechanism in the water wall tubes of power plants.

## 2. Experimental Method

### 2.1. Microstructural Characterization

The water wall tube is commercial low-alloy ASTM SA-213 T12 steel, whose composition is given in Table 1.

**Figure 1:**
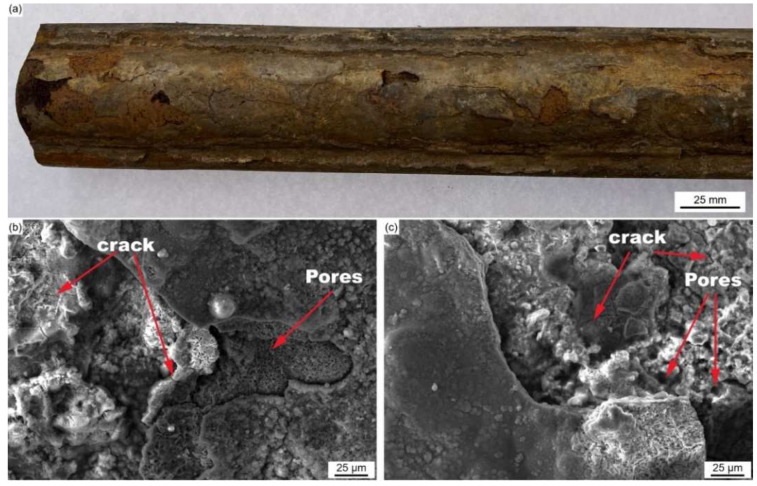
Macro- (**a**) and micro-morphologies (**b**,**c**) of the in-furnace water wall tube.

Figure 1a shows the macro- and micro-surface morphologies of the in-furnace water wall tube.

**Figure 2:**
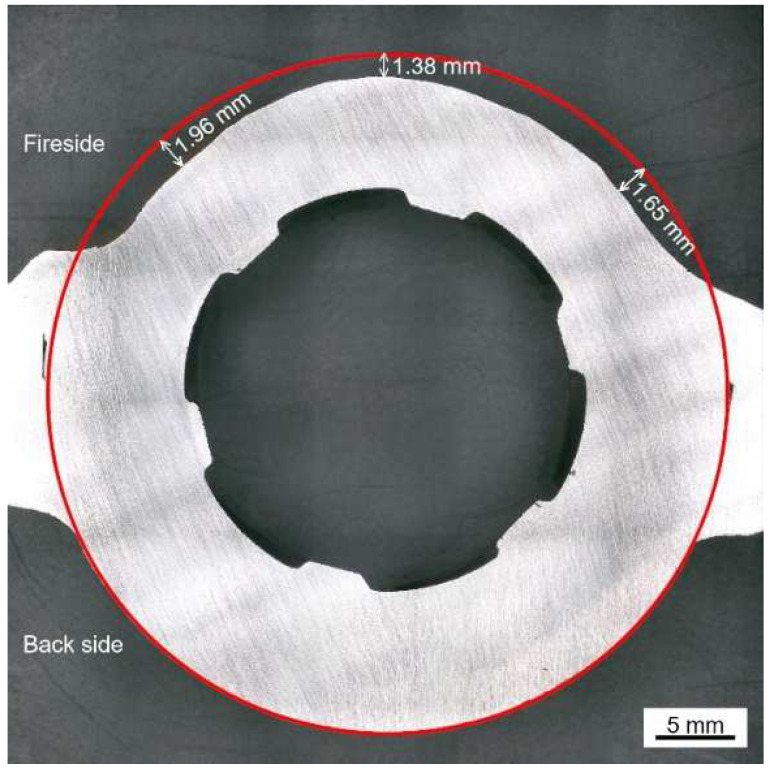
Cross-sectional macro-morphology of the water wall tube.

The fireside of the water-cooled wall was covered with brownish adherents, which were multi-layered and poorly bonded. Figure 1b,c show that the surface precipitations consist of cracks, porous holes, as well as needle-shaped or spherical particles scattering over the rugged pits and flat layer. Figure 2 shows the cross-sectional macro-morphology of the water wall tube after the removal of the surface adherents.

As shown in Figure 2, significant and uneven wall thinning (1.5–2.0 mm) of the water wall tube was observed on the fireside.

To obtain more details, scanning electron microscopy (SEM, VEGA 3XMU TESACAN, Brno, Czech Republic), equipped with a backscattered electron (BSE) detector and electron probe microanalyzer (EPMA, EPMA-1720 Shimadzu, Kyoto, Japan), was used to observe the morphologies and chemical compositions of the adherents on fireside of the water wall tubes, and transmission electron microscopy (TEM, JEM2100 JEOL, Tokyo, Japan) was used to observe the microstructure of the water wall tubes after long-term service in the furnace.

### 2.2. Laboratory Experiment Setup

**Figure 3:**
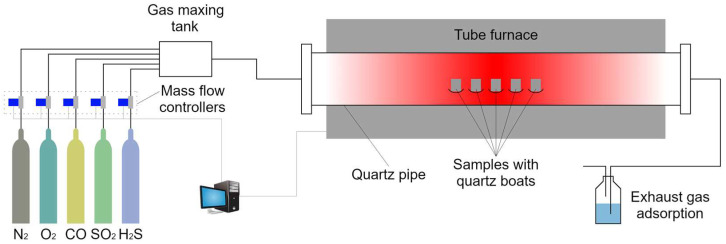
Schematic diagram of the high-temperature fireside corrosion apparatus.

Figure 3 shows the schematic diagram of the laboratory-scale high-temperature fireside corrosion apparatus, which is mainly composed of a gas supply system, a high-temperature tube furnace, and an exhaust gas treatment unit.

The flow rate of each experimental gas was controlled by the mass flow controller. Then, all of the gases entered a gas mixing tank to be fully mixed. The flow rate of the mixed gas was maintained at 100 mL/min. The mixed gas was reacted with experimental specimens in a high-temperature tube furnace. Finally, the reacted gas was absorbed by NaOH solution in the exhaust gas treatment unit. This laboratory experiment was implemented to obtain the short-term simulated corrosion sample for the composition identification and corrosion kinetics characterization. To improve the common adaptation of the corrosion experimental results, SA-213 T12 steel was selected to carry out the fireside corrosion experiment. An SA-213 T12 steel tube was cut into specimens 10 mm × 10 mm × 3 mm. All of the specimens were ground up to a 1000-grit finish with silica papers, ultrasonically cleaned in ethanol and deionized water, and dried at 50 °C for 24 h in an oven. Finally, the surface area and original weight of each specimen were measured by a vernier caliper and a balance, respectively.

With the aim of studying the corrosion behavior of the water wall tubes, a reducing atmosphere with a high concentration of CO was simulated in this paper, as shown in Table 2. In the combustion zone of the low-NOx combustion boilers, the CO concentration ranged from approximately 10,000 ppm (1%) to 50,000 ppm (5%), and the H_2_S ranged from approximately 1000 ppm (0.1%) to 5000 ppm (0.5%). Therefore, the upper limits of 5% for CO and 0.5% for H_2_S were selected for the reducing atmosphere in the laboratory simulation experiment. This consideration allowed for the simulation and evaluation of the most severe operating conditions. Above the critical pressure of the water/steam, only single-phase water or steam exists in the boiler water-cooled wall. The working fluid temperature ranged from 340 °C to 440 °C. Therefore, a metal temperature of 400 °C was selected for the laboratory simulation experiment, as this represents the realistic average temperature of the water-cooled wall during the boiler operation.

On the sample surface, there were two conditions, including no ash coating and real coal ash coating. The component of real coal ash provided by the power plant is shown in Table 3.

After every period of the corrosion experiment, the weight of the specimens was measured to calculate the weight-gain data. To ensure the accuracy of the weight-gain data, three parallel specimens were prepared for each corrosion period. After the high-temperature fireside corrosion experiment, the morphologies and chemical compositions of the corrosion products on the specimens were characterized by using SEM and EPMA. The phase of the corrosion products was detected by X-ray diffraction (XRD, D8 advance).

## 3. Results

### 3.1. Furnace Samples

**Figure 4:**
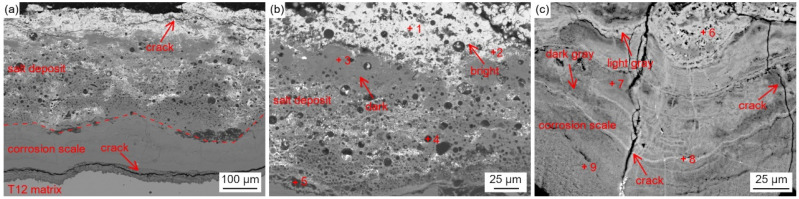
Cross-sectional BSE images of the in-furnace water wall tubes: (**a**) the entire adhesions, (**b**) the salt deposit, and (**c**) the corrosion scale.

Figure 4 shows the cross-sectional micro-morphology of the water wall tube on the fireside.

As shown in Figure 4a, the adherents on the surface of the water wall tubes can be divided into two parts, which include the salt deposit with a loose and porous structure and the corrosion scale with a relatively dense structure. The cracks and pores in the salt deposit and corrosion scale can act as diffusion pathways for corrosive gases to diffuse inwards. Discontinuous black island-like products were observed near the interface of the salt deposit and corrosion scale. Meanwhile, significant delamination was observed in both the salt deposit and corrosion scale, as shown in Figure 4b,c.

**Figure 5:**
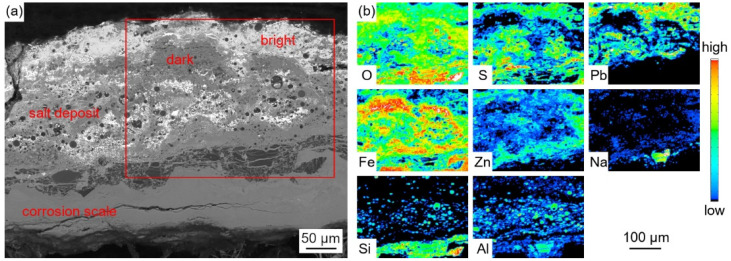
Cross-sectional BSE images (**a**) and EPMA elemental distribution maps (**b**) of the salt deposit.

Figure 5 shows the EPMA elemental distribution maps of the salt deposit.

The enrichment of the Pb element was observed in the bright region, while the enrichment of Fe and Zn was observed in the dark region. The distribution characteristic of the S element was basically similar with that of Pb and Zn, which proved the formation of Pb and Zn sulfides. The distributions of O and S in the Fe-enrich region proved the formation of Fe oxides and sulfides. In addition, the enrichment of Al, Si, and O was observed in the spherical particles and black island-like products, which proved the formation of silicates or aluminosilicates.

It has been widely reported that the salt deposits formed under oxy-fuel combustion consisted of silicates/aluminosilicates [20], sulfates [21] (CaSO_4_, Na_2_SO_4_, K_2_SO_4_, etc.), and chlorides [22,23] (NaCl, KCl, etc.), which seemed to be quite different from the results obtained in this paper. Oxidizing and reducing atmospheres close to the water wall tubes were changed alternately inside of the coal-fired plant after the low-combustion modification. The volatility proportion of trace elements, such as Zn and Pb, was higher in reducing atmosphere than that in the oxidizing atmosphere [24]. As a result, more gaseous Zn-/Pb-containing species reacted with H_2_S and condensed onto the surfaces of the deposit in the reducing atmosphere. These Zn/Pb sulfides would affect the diffusion of H_2_S- or S-containing species into the deep side of corrosion product, which may accelerate the corrosion rate [25,26].

**Figure 6:**
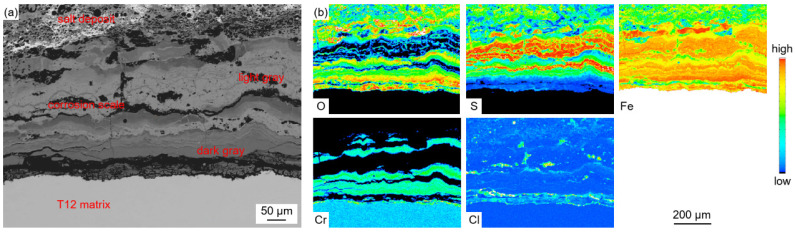
Cross-sectional BSE images (**a**) and EPMA elemental distribution maps (**b**) of the corrosion scale.

Figure 6 shows the EPMA elemental distribution maps of the corrosion scale.

The enrichment of the Fe element was observed in the whole profile section of the corrosion scale. The enrichment of S and O was observed in the light-gray parts and dark-gray parts, respectively. This indicated that the corrosion scale was composed of Fe oxides and sulfides. The distribution of Cr was found in the O-enriched region, which proved the formation of Fe-Cr spinel oxides. The distribution of S and O did not coincide with each other, which indicated the competing relationship between sulfation and oxidation during the corrosion process. Trace amounts of the Cl element were also observed in the corrosion scale. During combustion, Cl-containing species in coal and other inorganic salts deposited on the surface of the water wall tubes, forming low-melting-point co-crystals. These co-crystals diffused inwards along the cracks and pores in the salt deposit and corrosion scale.

Five points in Figure 4b and four points in Figure 4c were selected to contrast their compositions by EPMA and presented in Table 4. The results show that the salt deposit contained heavy metal elements such as Pb, Zn, Sn, Sb, and so on, due to the direct condensation of metal vapors under the steep temperature gradient generated during combustion. The bright region was mainly composed of Pb sulfides, while the dark region can be divided into two parts. The parts represented by point 2 contained Pb and Zn sulfides, as well as Fe oxides and sulfides, and the parts represented by point 3 contain mainly Fe oxides and sulfides.

The corrosion scale mainly contained Fe, O, S, and Cr. The light-gray parts (represented by point 6 and point 8) contained Fe sulfides, and the dark-gray parts (represented by point 7 and point 9) contained Fe oxides and small amounts of Fe sulfides and Cr oxides.

**Figure 7:**
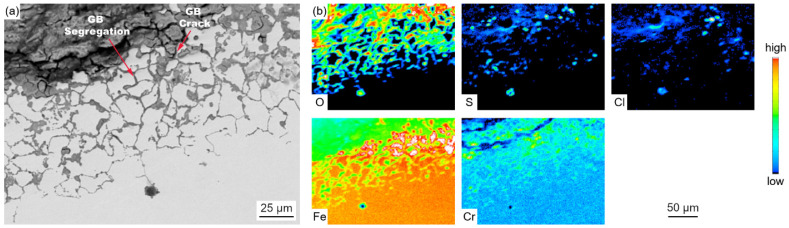
Cross-sectional BSE images (**a**) and EPMA elemental distribution maps (**b**) of the metal substrate layer.

Figure 7 presents the elemental distribution map of metal substrates adjacent to the oxidation layer.

The BSE image magnifies the details of GB precipitation, segregation, and cracks, and the elemental map illustrates the sulfur and chlorine inward transportation and diffusion, and the enrichment of chromium at the GB precipitation phase.

**Figure 8:**
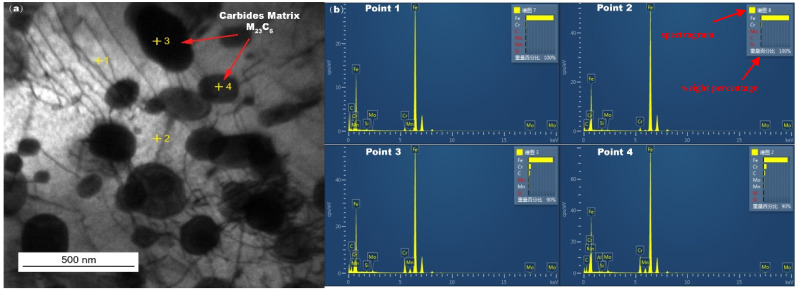
(**a**,**b**) TEM image of the in-furnace water wall tube.

Figure 8 shows the TEM image of the in-furnace water wall tubes sampled at metal substrates after a long period of service, and grain boundary (GB) precipitated phases were found in the matrix of the water wall tubes. These precipitated phases were identified in Table 5 as the carbide matrix M_23_C_6_ by EDS, which is mainly located in the vicinity of the GBs.

### 3.2. Laboratory Samples

**Figure 9:**
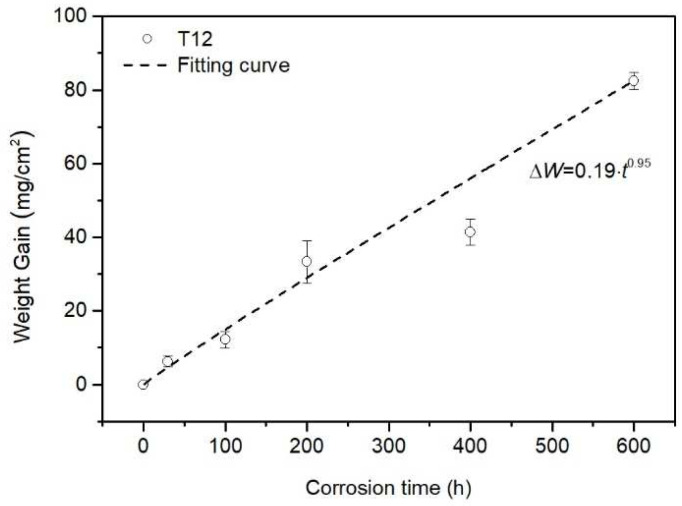
Kinetic curve of the SA-213 T12 steel with no ash coating after exposure to the reducing atmosphere at 400 °C.

Figure 9 shows weight-gain data and corrosion kinetic curve of the SA-213 T12 steel with no ash coating after exposure to the reducing atmosphere at 400 °C.

The weight-gain data increased with the exposure time. The corrosion kinetics can be fitted by the following equation:

(1) $$\text{∆}W=k_{p}t^{n}$$

where Δ*W* is the weight change in the specimen (mg/cm^2^), *n* is the time exponent that describes the time dependence of oxide growth, *k_p_* is the corrosion rate constant (mg/(cm^2^h^n^)), and t is the exposure time (h). The values of *k_p_* and *n* obtained from the corrosion experiment were 0.19 and 0.95, respectively. This indicates that the corrosion kinetics of the SA-213 T12 steel in the reducing atmosphere at 400 °C followed the linear law. This result agrees with the result reported by Xu et al. [27]. Therefore, it can be deduced that the corrosion scale formed in the reducing atmosphere with CO and H_2_S is nonprotective and cannot increase the difficulty of mass transfer. They also found that the corrosion rate of specimens in presence of CO was higher than that in absence of CO, because CO destroyed the formation of oxides that possessed slight corrosion resistance. In addition, the corrosion kinetics of 12Cr1MoV steel in an oxidizing atmosphere with SO_2_ followed a parabolic law, as reported by Xu et al. [27]. This difference between corrosion kinetics in a reducing atmosphere and an oxidizing atmosphere suggests that CO and H_2_S can significantly promote corrosion.

**Figure 10:**
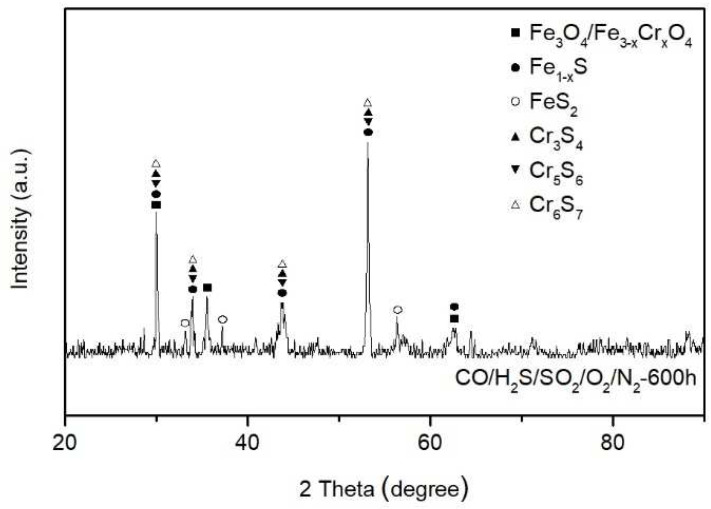
XRD analysis of SA-213 T12 steel with no ash coating after exposure to the reducing atmosphere at 400 °C for 600 h.

Figure 10 shows the XRD pattern of SA-213 T12 steel with no ash coating after exposure to the reducing atmosphere at 400 °C for 600 h.

The diffraction results show that corrosion products were primarily composed of magnetite oxides (Fe_3_O_4_), spinel oxides (Fe_3−*x*_Cr*_x_*O_4_, where *x* is the stoichiometric coefficient), Fe sulfides (FeS_2_ and Fe_1−*x*_S), and Cr sulfides (Cr_3_S_4_, Cr_5_S_6_, and Cr_6_S_7_).

**Figure 11:**
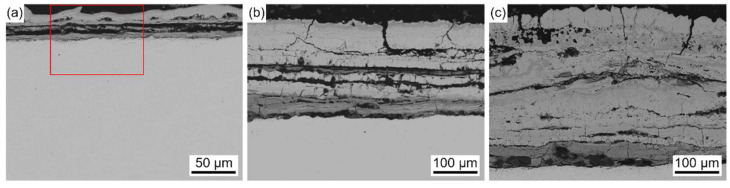
Cross-sectional BSE images of the corrosion scale on SA-213 T12 steel with no ash coating after exposure to the reducing atmosphere at 400 °C for different times: (**a**) 30 h, (**b**) 400 h, and (**c**) 600 h.

Figure 11 shows the cross-sectional micro-morphology of the corrosion scale on SA-213 T12 steel with no ash coating after exposure to the reducing atmosphere at 400 °C for different times.

As shown in Figure 11, the corrosion scale showed a double-layer structure. The outer layer was highly loose, with a large number of internal holes and cracks, and the thickness was approximately 160 μm, while the inner layer was relatively dense, and the thickness was approximately 45 μm. Transverse cracks were observed in the outer layer and near the interface of the outer layer and inner layer, which led to the spalling of the corrosion scale. In addition, cracks and pores in both the outer and inner layers can act as conduits for corrosive gases to diffuse inwards, increasing the corrosion rate and ultimately leading to a linear law of corrosion kinetics.

**Figure 12:**
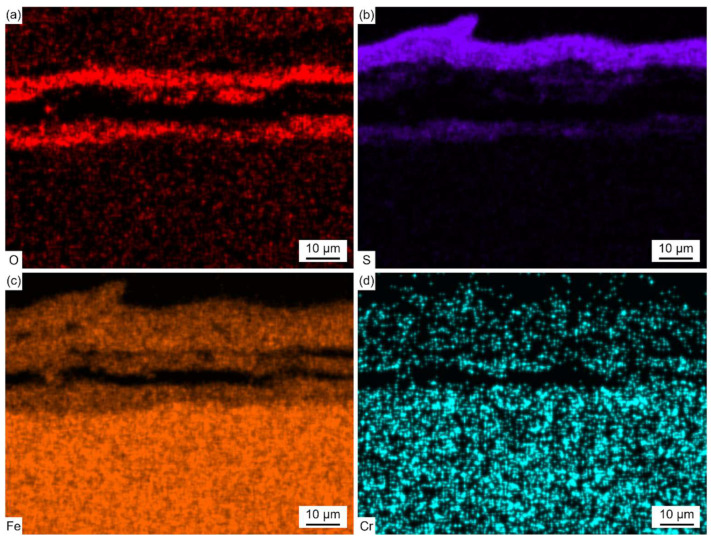
(**a**–**d**) EDS elemental distribution maps of the corrosion scale corresponding to the region (solid line) in Figure 11a.

Figure 12 shows the EDS elemental distribution maps of the corrosion scale corresponding to the region (marked with the solid line) in Figure 11a.

Fe and S were found in the outer layer, and Fe, Cr, O, and a trace amount of S were found in the inner layer. The thickness of the outer layer was comparable to that of the inner layer after exposure to the reducing atmosphere at 400 °C for 30 h. However, the thickness of the outer layer was much thicker than that of the inner layer, as shown in Figure 11b,c. Thus, the growth rate of the outer layer was more rapid than that of the inner layer with the increase in the exposure time. This was due to the fact that, in the reducing atmosphere with a high concentration of CO, the oxidation process was strongly inhibited and sulfation predominantly took place.

**Figure 13:**
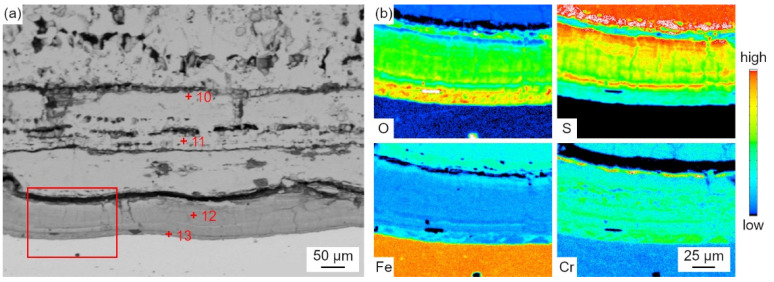
Cross-sectional BSE images (**a**) and EPMA elemental distribution maps (**b**) of the corrosion scale on SA-213 T12 steel with no ash coating after exposure to the reducing atmosphere at 400 °C for 600 h.

Figure 13 shows the cross-sectional micro-morphology and EPMA elemental distribution maps of the corrosion scale on SA-213 T12 steel with no ash coating after exposure to the reducing atmosphere at 400 °C for 600 h.

As shown in Figure 13b and Table 6, the outer layer contained Fe and S, and the inner layer contained Fe, Cr, O, and S.

The distribution of Cr was in a layer form and the distribution of S was in a root-like form. The enrichment of O was near the interface of the corrosion scale and matrix. The enrichment of S and O did not coincide with each other as well. The characteristic distribution of S in the inner layer may be related to the inward diffusion of gas molecules. During the corrosion process, CO and H_2_S diffused inward along fast diffusion paths, like grain boundaries and cracks, in the inner layer. Then, some of the oxides near these paths directly reacted with H_2_S to form Fe sulfides, and some were first reduced by CO and then further reacted with H_2_S to form Fe sulfides. So, the distribution of S in the inner layer is basically similar with the inward diffusion paths of CO and H_2_S.

Combined with the XRD results in Figure 10, it was confirmed that the outer layer was composed of Fe_1−*x*_S and FeS_2_, and the inner layer was composed of sulfides, including Fe_1−*x*_S, FeS_2_, Cr_3_S_4_, Cr_5_S_6_, and Cr_6_S_7_, and oxides, including Fe_3_O_4_ and Fe_3-*x*_Cr*_x_*O_4_. Due to the poor toughness of the sulfides formed in the outer layer, a large number of cracks were generated in the outer layer. Figure 13 shows that the thicker outer corrosion scale layer is mainly composed of Fe sulfides, while the thinner inner corrosion layer is primarily composed of spinel oxides and magnetite oxides. It can be inferred that the presence of spinel oxides and magnetite oxides can further inhibit the inward transport and diffusion of S, while not inhibiting the outward diffusion of Fe. This characteristic of allowing easy cation transport while forming a diffusion barrier for anions causes the outer corrosion layer to grow faster and thicker, while the inner corrosion layer grows at a slower rate and is relatively thin.

**Figure 14:**
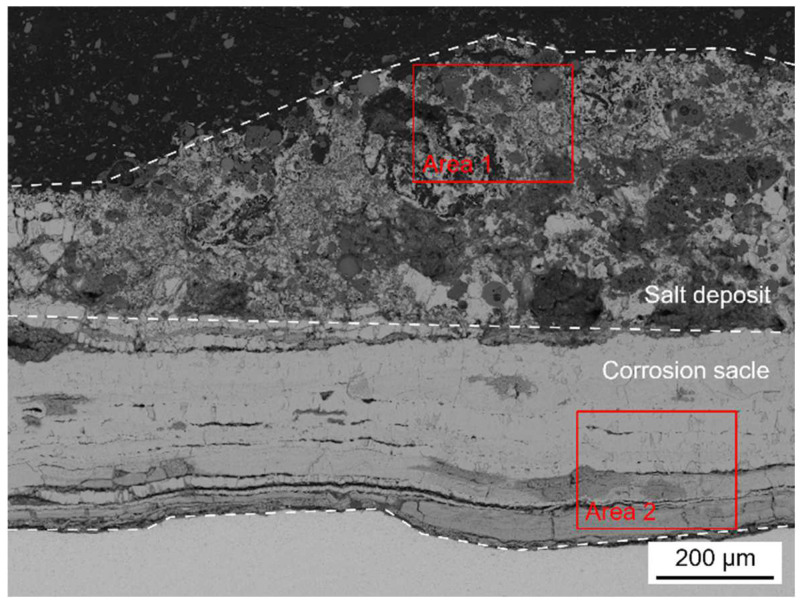
Cross-sectional BSE images of the corrosion scale on SA-213 T12 steel with real coal ash coating after exposure to the reducing atmosphere at 400 °C for 600 h.

Figure 14 shows the cross-sectional micro-morphology of the corrosion scale on SA-213 T12 steel with a real coal ash coating after exposure to the reducing atmosphere at 400 °C for 600 h. The adherents on the surface of the SA-213 T12 steel can be divided into two parts, the salt deposit with a loose and porous structure and the corrosion scale with a relatively dense structure, which was similar to the adherents found on the surface of the furnace samples in Figure 4.

**Figure 15:**
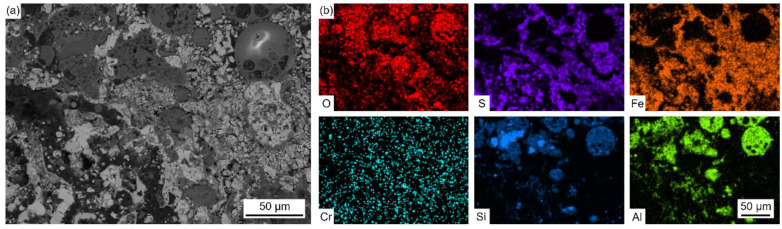
Cross-sectional BSE images (**a**) and EDS elemental distribution maps (**b**) of the salt deposit corresponding to Area 1 in Figure 14.

**Figure 16:**
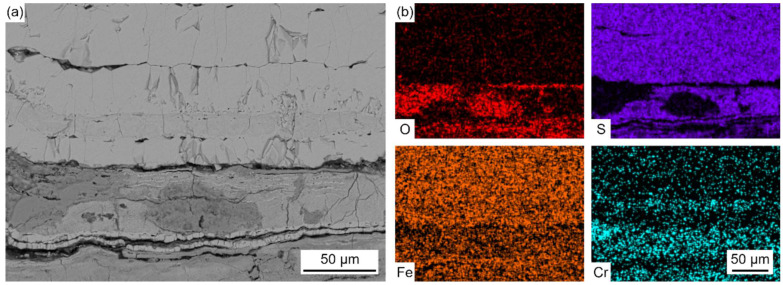
Cross-sectional BSE images (**a**) and EDS elemental distribution maps (**b**) of the corrosion scale corresponding to Area 2 in Figure 14.

Figure 15 and Figure 16 show the cross-sectional BSE images and EDS elemental distribution maps of the salt deposit and corrosion scale in Figure 14.

As shown in Figure 15, the salt deposit mainly contained Fe oxides and sulfides. The distribution of S and O did not coincide with each other as well. In addition, the enrichment of Al, Si, and O was observed in the spherical particles, which proved the formation of silicates or aluminosilicates. Compared with the results in Figure 5, no heavy metal elements (such as Pb, Zn, Sn, and Sb) were found in the salt deposit formed on the laboratory samples with the real coal ash coating. This indicates that these elements were produced during the combustion of coal. The results of the laboratory samples with the real coal ash coating in Figure 15 reveal the presence of distinct spheroidal Al-Si oxides within the salt deposits, which are consistent with the shape and distribution shown in Figure 5. These Al-Si oxides are absent from the salt deposits in the results of the laboratory samples with no coal ash coating, as shown in Figure 11. The relatively loose surroundings of these oxides provide highly favorable conditions for elemental transportation and diffusion. As shown in Figure 16, the corrosion scale showed a double-layer structure. The outer layer contained Fe and S and the inner layer contained Fe, Cr, O, and S, which was similar to the results obtained in Figure 12 and Figure 13. The corrosion rate of the samples with real coal ash coating (~90 mg/cm^2^) was higher than that of the samples with no ash coating (~80 mg/cm^2^), which indicates that coal ash can promote the process of corrosion.

## 4. Discussion

There is no doubt that the initiation of the corrosion is induced by the presence of the high-temperature, corrosive environment. The oxidation and sulfurization process is necessitated by the transportation and diffusion of O, S, Fe, and Cr, and the driving force for this step underlies in the chemical potential and concentration gradient by Fick’s Law [28]. The role of trace elements in the corrosion growth and propagation remained unclear. Song [29] reported that chlorine facilitated the kinetic rate of Fe_3_O_4_ growth, while Schoell [11] believed that chlorides in the salt deposition made it easy for surface pitting to lead to internal cracks. It was observed in Figure 6 that chlorine precipitates in the transition layer between the oxidation layer and metal substrates, where the crack is formed. The trace elements Pb and Zn did not diffuse inward as far as Cl, which was embedded only in the salt deposit layer; this is explained by trace heavy elements being susceptible to capture by sulfates to form low-melting-point compounds [30], as well as having an assistant role in accelerating the diffusion rate. GB precipitation, segregation, and cracks, as revealed by Figure 7, have a significant role in sensitization in the corrosion process. Oxidation occurs evenly adjacent to metal substrates, and GB segregation preferably complies with the chromium enrichment. The effect of the segregation of the GBs is the attenuation deep into the metal substrates, which provides strong evidence for the intergranular corrosion attack. Carbide matrix precipitation on the GB was identified by TEM characterization.

The inhomogeneous distribution of the carbide matrix on the GB decreased the fracture toughness of this region and made the GB more susceptible to cracks [31]. With the rapid development of corrosion layer growth, the possibility of embrittlement and dilation between the corrosion layer and metal substrates becomes more likely. With the synergetic impact of inner tube stress, the exfoliation of the corrosion layer will inevitably occur, leading to the corrosion thinning of the tube thickness or tube burst failure.

Laboratory experiments revealed that the reduced atmosphere corrosion kinetic simulation followed the linear law. The EPMA and XRD characterization presented a different corrosion scale structure layer in comparison to the furnace sample. Figure 16 presents a relatively shallow, uniform distribution without delamination, a corrosion scale layer with Fe sulfides dominating in the outer layer, and Cr sulfides, magnetite oxides, and spinel oxides prevailing in the inner layer. Furnace sample microscopic observations indicate a deeper thickness corrosion scale layer, with Fe sulfides and Fe oxides forming a staggered layout in the outer layer, and an unevenly thinner thickness corrosion layer with a delaminated distribution of Fe-Cr spinel oxides, as shown in Figure 7b. Under a pure reduced atmosphere, the uniform and consistent distribution of the corrosion layer without delamination indicates that the atmospheric conditions have a significant impact on the formation of the corrosion layer. Fe-Cr oxides dominate under an oxidizing atmosphere, while Fe sulfides dominate under a reduced atmosphere. The frequent shift between the oxidizing and reducing properties of the atmosphere around the water wall tubes during boiler operation may contribute to the delaminated oxidation layer.

**Figure 17:**
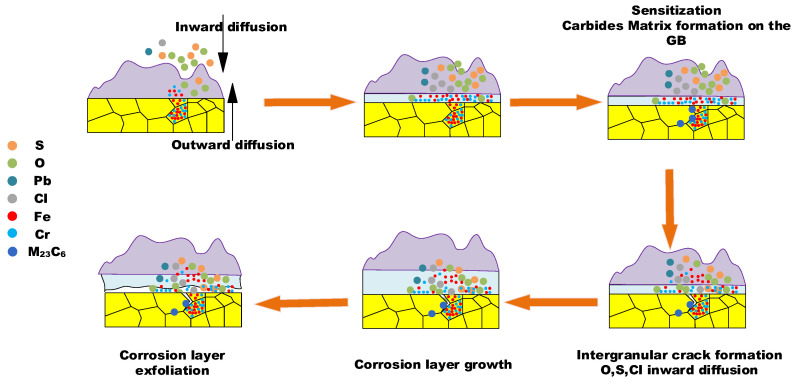
The fireside corrosion failure mechanism of the water wall tube.

The corrosion failure mechanism is depicted in Figure 17 and can be split into the following three main steps: (1) the initial step is the chemical potential- and concentration-governed inward and outward transportation and diffusion, including the oxidation layer formation and salt scale deposit; (2) the second step is the development of sensitization at the GB and carbide matrix formation, including GB segregation and the intergranular attack of O, S, and Cl, through the pathway of the GB; (3) the third step is corrosion layer growth and exfoliation, including kinetic-governed growth and crack dilation-induced tube thickness thinning. It is worth noting that the results of the first and second mechanism steps are unequivocally observed and confirmed by the microstructure characterization of the water wall tubes sampled from the furnace. However, the third step is not directly associated with microscopic observation. It is derived from the macro-morphology observation of the furnace sample, and is supposed to be the important step accounting for the pending corrosion failure.

## 5. Corrosion Mitigation Actions

The severely corroded, thinning tubes were replaced with new SA-213 T12 tubes during the shutdown activities. All of the new tubes were subsequently coated with NiCrTi alloy powder using the High-Velocity Oxygen Fuel (HVOF) spray method as well as Arc Spray to improve the resistance to corrosion failure. Laboratory corrosion kinetic tests under a reducing atmosphere dominated by CO and H_2_S show that a reducing atmosphere accelerates the corrosion rate of the tubes. Therefore, in actual power plant operation, it is necessary to minimize the possibility of a reducing atmosphere occurring. Real-time monitoring and control of the reducing atmosphere is a problem that needs further consideration. In consideration for the above-mentioned water wall tube operation atmosphere optimization, the following two actions were implemented: (1) bell-shaped conical shells were installed into all lateral burner air intakes, which was expected to enrich the oxygen concentration near the lateral water wall, ensuring better combustion and reducing the potential for a corrosion environment; (2) one set of real-time gas analyzers for CO and H_2_S were installed to monitor the water wall atmosphere environment, which can be targeted and controlled in the coordinated control system (CCS) of the unit.

## 6. Conclusions

In this paper, the corrosion thinning behavior of the water wall tubes of an ultra-supercritical power plant was investigated via SEM, EPMA, XRD, TEM, and laboratory simulation experiments. The following points are concluded:

Laboratory experiments revealed the reduced atmosphere corrosion kinetic simulation followed the linear law. The EPMA and XRD characterization presented a different corrosion scale structure layer in comparison to the furnace sample, while the reduced atmosphere condition in the laboratory experiment inhibited the oxidation process and layer growth. The frequent shift between the oxidizing and reducing properties of the atmosphere around the water wall tubes during boiler operation may contribute to the delaminated oxidation layer.

The fireside corrosion thinning mechanism is summarized into the following three main steps: (1) the initial step is the chemical potential- and concentration-governed inward and outward transportation and diffusion, including the oxidation layer formation and salt scale deposit; (2) the second step is the development of sensitization at the GB and carbide matrix formation, including the GB segregation and intergranular attack of O, S, and Cl, through the pathway of the GB; (3) the third step is corrosion layer growth and exfoliation, including kinetic-governed growth and crack dilation-induced tube thickness thinning.

## Figures and Tables

**Table 1:** Component of water wall tube materials (wt.%).

	C	Si	Mn	P	S	Cr	Mo	Fe
SA-213 T12	0.130	0.270	0.550	0.004	0.008	1.060	0.470	Bal.

**Table 2:** Experimental conditions.

Types of Samples	Gas Composition (%)					Temperature (°C)	Exposure Time (h)
	CO	O_2_	SO_2_	H_2_S	N_2_		
no ash coating	5	0.1	0.1	0.5	94.3	400	30, 100, 200, 400, 600
real coal ash coating	5	0.1	0.1	0.5	94.3	400	600

**Table 3:** Component of real coal ash (wt.%).

SiO_2_	Fe_2_O_3_	Al_2_O_3_	CaO	MgO	SO_3_	TiO_2_	K_2_O	Na_2_O	P_2_O_5_	MnO_2_
52.16	4.98	15.83	6.38	2.64	4.65	0.82	1.91	2.57	0.08	0.08

**Table 4:** Chemical component for the points selected in Figure 4.

No.	Elements														
	C	O	S	Fe	Pb	Zn	Ca	K	Al	Si	Cr	Mo	Mn	Sn	Sb
1	3.54	3.66	16.17	1.06	70.28	0.68	0.20	0.11	-	-	-	-	-	3.71	0.59
2	4.46	16.27	4.08	34.20	4.95	20.57	0.15	0.22	3.20	5.47	-	1.86	-	3.06	1.61
3	3.31	16.25	4.15	73.92	1.40	0.42	0.46	0.09	-	-	-	-	-	-	-
4	2.66	44.75	0.62	2.85	3.42	0.41	0.85	0.85	17.57	26.02	-	-	-	-	-
5	3.24	33.1	1.73	3.52	1.37	2.65	0.42	0.72	1.45	51.79	-	-	-	-	-
6	2.62	-	28.67	64.36	-	-	0.04	-	-	0.73	2.23	0.74	0.62	-	-
7	3.29	11.28	2.64	78.04	-	-	-	-	-	0.69	2.44	1.3	0.33	-	-
8	2.98	-	23.31	69.02	-	-	0.17	-	-	0.76	2.71	0.57	0.48	-	-
9	3.72	12.35	2.99	77.17	-	-	0.03	-	-	0.72	2.24	0.77	-	-	-

**Table 5:** Atomic percentage for the points selected in Figure 8.

	Atomic Percentage %			
	Point 1	Point 2	Points 3	Point 4
C	6.24	2.79	18.91	18.02
Al				0.55
Si	0.59	0.76	0.4	0.36
Cr	2.25	2.35	7.54	8.77
Mn	0.31	0	1.51	1.69
Fe	90.23	93.66	70.68	69.52
Mo	0.38	0.43	0.96	1.09
Matrix (Cr, Mn, Fe, Mo)	93.17	96.44	80.69	81.07
Carbide Matrix			25.60	26.99

**Table 6:** Chemical components for the points selected in Figure 13a.

No.	Elements							
	C	O	S	Fe	Cr	Si	Mn	Mo
10	9.31	-	33.16	57.54	-	-	-	-
11	10.65	-	31.05	58.31	-	-	-	-
12	12.33	4.06	22.17	56.45	2.72	0.71	0.60	0.95
13	14.75	6.11	11.58	61.98	2.94	0.89	0.67	1.08

## Data Availability

The original contributions presented in this study are included in the article. Further inquiries can be directed to the corresponding author.

## Nomenclature

USC	ultra-supercritical
HTHC	high-temperature hot corrosion
LTHC	low-temperature hot corrosion
SEM	scanning electron microscopy
BSE	backscattered electron
TEM	transmission electron microscopy
EPMA	electron probe microanalyzer
XRD	X-ray diffraction
Greek symbols	
∆	changeable quantity
Mathematical symbols	
*W*	weight change in the specimen, mg/cm^2^
*k*	corrosion rate constant, mg/(cm^2^h^n^)
*t*	exposure time, h
Subscripts and superscripts	
*p*	specific component
*n*	exponential time regression value
